# The Musculoskeletal Involvement After Mild to Moderate COVID-19 Infection

**DOI:** 10.3389/fphys.2022.813924

**Published:** 2022-03-18

**Authors:** Patty K. dos Santos, Emilly Sigoli, Lorenna J.G. Bragança, Anabelle S. Cornachione

**Affiliations:** Muscle Physiology and Biophysics Laboratory, Department of Physiological Sciences, Federal University of São Carlos (UFSCar), São Carlos, Brazil

**Keywords:** COVID-19, musculoskeletal system, mild to moderate COVID-19, SARS-CoV-2, long COVID, non-hospitalized individuals, muscle symptoms

## Abstract

COVID-19, a disease caused by the novel coronavirus SARS-CoV-2, has been drastically affecting the daily lives of millions of people. COVID-19 is described as a multiorgan disease that affects not only the respiratory tract of infected individuals, but it has considerable effects on the musculoskeletal system, causing excessive fatigue, myalgia, arthralgia, muscle weakness and skeletal muscle damage. These symptoms can persist for months, decreasing the quality of life of numerous individuals. Curiously, most studies in the scientific literature focus on patients who were hospitalized due to SARS-CoV-2 infection and little is known about the mechanism of action of COVID-19 on skeletal muscles, especially of individuals who had the mild to moderate forms of the disease (non-hospitalized patients). In this review, we focus on the current knowledge about the musculoskeletal system in COVID-19, highlighting the lack of researches investigating the mild to moderate cases of infection and pointing out why it is essential to care for these patients. Also, we will comment about the need of more experimental data to assess the musculoskeletal manifestations on COVID-19-positive individuals.

## Introduction

Coronavirus Disease 2019 (COVID-19), a disease caused by the novel coronavirus SARS-CoV-2 (Severe Acute Respiratory Syndrome Coronavirus 2), has been drastically affecting and changing people’s lifestyle around the world since 2020. It caused a social and economic global crisis, leading to the collapse of public health systems in various countries. COVID-19 has demonstrated to affect a multi-variety of organs, including the musculoskeletal system, causing symptoms such as fatigue, arthralgia, myalgia and muscle weakness, which can persist during weeks or months after the end of the infection, affecting the daily lives of numerous individuals named as “long haulers” (Jacobs et al., 2020; Sagarra-Romero and Viñas-Barros, 2020; Aiyegbusi et al., 2021; Akbarialiabad et al., 2021).

Interestingly, the majority of the reports in the scientific literature focus on the musculoskeletal symptomatology and on the severe and critical forms of COVID-19, comprising studies with hospitalized patients who needed ventilator support throughout the disease course. Little is known about the individuals who had the mild to moderate forms of the infection, and whose musculoskeletal symptoms can persist. Therefore, the aim of this narrative review is to point out the current evidence on the musculoskeletal aspects of the SARS-CoV-2 infection, highlighting and commenting about the lack of information and experimental data regarding the musculoskeletal manifestations in mild to moderate COVID-19 cases (non-hospitalized individuals).

We conducted a comprehensive literature search on five electronic databases, namely Google Scholar, CAPES Periodicals, PubMed, ScienceDirect and Virtual Health Library (VHL) Regional Portal. The search terms used were “COVID-19”, “musculoskeletal”, “musculoskeletal symptoms”, “muscle”, “muscle weakness”, “myalgia”, “fatigue”, “creatine kinase”, “long-hauler”, “long-COVID-19”, “mild to moderate COVID-19”, and “mild COVID-19”, combined using the Boolean operator AND. We included original research articles, brief reports, case reports, case series, short communications, reviews, mini reviews, editorials, features, letters to the editor, pre-proof and in press articles published from December 2019 to July 2021, and written in English or Portuguese. Articles in the form of preprint manuscripts, correspondence, opinion, perspectives and insights were excluded. Studies were considered eligible for reading and inclusion if their focus was the musculoskeletal system (muscle pathophysiology, persistent muscle symptoms, laboratory findings related to the musculoskeletal system, musculoskeletal sequelae). All the information were synthesized and discussed in a narrative manner in each section within this review.

## Background

### Origin and Virology of COVID-19

The novel coronavirus SARS-CoV-2 emerged in December 2019 as a cluster of “pneumonia of unknown etiology” epidemiologically linked to a seafood market in Wuhan City, Hubei Province (People’s Republic of China) (World Health Organization, 2021a; Pan American Health Organization, 2021). Its genetic sequence was identified on 7 January 2020, sharing >95% of homology with the bat coronavirus and 79.5% sequence identity with the Severe Acute Respiratory Syndrome Coronavirus (SARS-CoV) (Abdelrahman et al., 2020; Lu et al., 2020; Rossi et al., 2020; Tang et al., 2020). The viral disease was named COVID-19 (Coronavirus Disease 2019) on 11 February 2020 and as the virus spread constantly and rapidly throughout the world, the World Health Organization (WHO) declared a pandemic situation on 11 March 2020 (World Health Organization, 2021a; Pan American Health Organization, 2021).

As of 23 August 2021, there have been 211.730.035 confirmed cases of COVID-19 worldwide, including 4.430.697 deaths (World Health Organization, 2021b). This number surpasses the total of deaths caused by both Middle Eastern Respiratory Syndrome (MERS; 2012-ongoing) and Severe Acute Respiratory Syndrome (SARS; 2002–2003) viral outbreaks (Ji et al., 2020; Wu and McGoogan, 2020). Fortunately, a total of 4.615.260.567 vaccine doses were already globally administered (World Health Organization, 2021b), which gives hope that better days will come.

**FIGURE 1:**
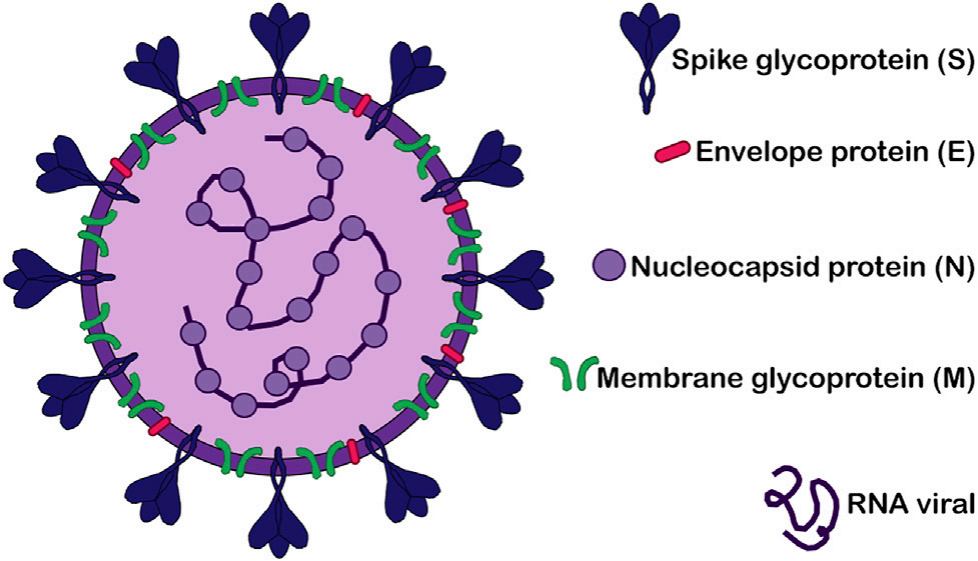
Structure of SARS-CoV-2. Schematic representation of SARS-CoV-2 virus structure highlighting its four major structural proteins: the spike glycoprotein (*S*), the envelope protein (*E*), the nucleocapsid protein (*N*) and the membrane glycoprotein (*M*). The RNA viral is also showed.

SARS-CoV-2 belongs to the *Betacoronavirus* genus (*Coronavirinae* family; *Nidovirales* order) and it is a positive-sense, single-stranded RNA virus with a diameter of approximately 60–140 nm (Disser et al., 2020; Machhi et al., 2020; Romagnoli et al., 2020; Chilamakuri and Agarwal, 2021). The virus structure is composed of four major structural proteins: (1) the spike glycoprotein (*S*), (2) the membrane glycoprotein (*M*), (3) the envelope protein (*E*), and (4) the nucleocapsid protein (*N*) (Disser et al., 2020; Tang et al., 2020; Chilamakuri and Agarwal, 2021) (Figure 1). The virus entry on the human cell is *via* the angiotensin-converting enzyme 2 (ACE2) surface receptor along with the transmembrane protease serine 2 (TMPRSS2). Briefly, the SARS-CoV-2 virus binds to ACE2 on the host cell surface (mainly alveolar epithelial cells), which leads to the proteolytic cleavage and the activation of the viral *S* protein by TMPRSS2. These processes expose a fusion peptide signal (S2 subunit of the *S* protein) that enables the fusion between the viral and the human membranes, and the final release of the viral RNA into the cell cytoplasm, where the virus will replicate using the host cell machinery (Bohn et al., 2020; Disser et al., 2020; Gonzalez et al., 2020; Tang et al., 2020).

## Susceptibility of Skeletal Muscle Cells to SARS-COV-2 Infection

Comprising 40% of the human body weight, the skeletal muscle is an important organized tissue composed by numerous bundles of fiber (myofibers) (Frontera and Ochala, 2015; Trovato et al., 2016; Mukund and Subramaniam, 2020). It has a crucial mechanical role, generating force and power through the conversion of chemical to mechanical energy, which yields movement, facilitating our daily activities. Furthermore, skeletal muscle can store energetic substrates (carbohydrates and amino acids) for the basal metabolism and it can contribute to heat production, stabilizing the body’s temperature (Frontera and Ochala, 2015; Trovato et al., 2016). Considering the multiple functions of the musculoskeletal system, essential to maintain a “healthy status,” and knowing that COVID-19 is a multi-organic disease that causes a large spectrum of symptoms (Baj et al., 2020; Gavriatopoulou et al., 2020; Gupta et al., 2020; Machhi et al., 2020; Singh et al., 2021), it was noteworthy to investigate the musculoskeletal susceptibility to SARS-CoV-2 infection.

**FIGURE 2:**
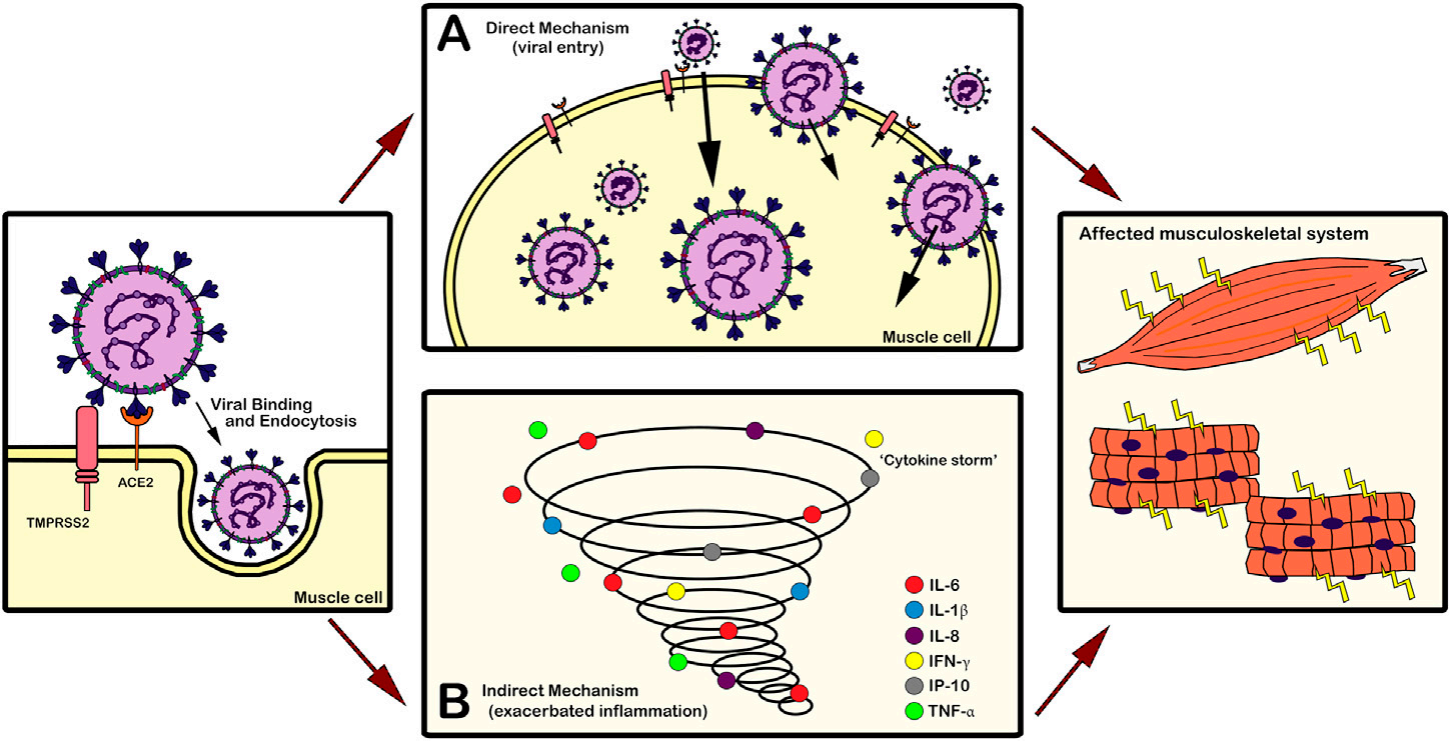
Mechanisms of action of SARS-CoV-2 on the skeletal muscle tissue. **(A)** Direct mechanism *via* SARS-CoV-2 binding to receptors on the skeletal muscle cell surface and entry of the virus into the cell. Several types of musculoskeletal cells express ACE2 and/or TMPRSS2, which allows this type of direct mechanism of action of the virus. **(B)** Indirect mechanism considering the adverse effects of the elevated inflammatory process (“cytokine storm”) caused by SARS-CoV-2 infection on the musculoskeletal tissue. The deregulated release of cytokines and chemokines (IL-6, IL-1β, IL-8, IFN-γ, IP-10, TNF-α) by the immune system results in exacerbated inflammation that can lead to multi-organ injuries. Both mechanisms **(A**,**B)** can affect the musculoskeletal system, causing manifestations such as fatigue and myalgia in symptomatic COVID-19 individuals. ACE2: angiotensin-converting enzyme 2; IFN-γ: interferon-gamma; IL-1β: interleukin-1β; IL-6: interleukin-6; IL-8: interleukin-8; IP-10: interferon-gamma inducible protein 10; TMPRSS2: transmembrane protease serine 2; TNF-α: tumor necrosis factor alpha.

There are two current hypotheses, proposed in the literature, suggesting different mechanisms of action of the virus on the skeletal muscle tissue (Figure 2). The first one considers a direct mechanism *via* SARS-CoV-2 binding to the ACE2 receptor on the skeletal muscle cell surface (Figure 2A) (Ferrandi et al., 2020). Studies showed that ACE2 is expressed in the skeletal muscle of mice, rats, and dystrophic mice, mainly in the cell sarcolemma (Fernandes et al., 2010; Takeda et al., 2013; Riquelme et al., 2014; Motta-Santos et al., 2016). However, Disser et al. demonstrated that only human smooth muscle cells and pericytes express ACE2. Using single-cell RNA sequencing of human data sets, they showed that human skeletal muscle cells, including satellite cells and myofibers, express only TMPRSS2 (Disser et al., 2020). This unexpected result leads us to hypothesize that maybe SARS-CoV-2 interacts with cells in an ACE-2 independent way and that the presence of TMPRSS2, which helps in the viral spike protein cleavage, together with other host cell receptors is sufficient to promote virus binding and infection. Indeed, Partridge et al. observed through flow cytometry that the SARS-CoV-2 *S* protein associates with multiple human epithelial cell lines without ACE2 (Partridge et al., 2021). However, they did not analyse any muscle cell line, which indicates that further studies are needed to elucidate if the susceptibility of the skeletal muscle tissue to COVID-19 can be directly *via* ACE2.

The second hypothesis proposes an indirect mechanism of action, considering the adverse effects of the elevated inflammatory process caused by SARS-CoV-2 infection on the musculoskeletal tissue (Figure 2B) (Ferrandi et al., 2020). The “cytokine storm,” a deregulated release of numerous cytokines by the immune system after SARS-CoV-2 infection in the lungs, results in exacerbated inflammation that can promote multi-organ injuries (Henderson et al., 2020; Ragab et al., 2020; Tay et al., 2020). Cytokines, such as interleukin-6 (IL-6), interleukin-1β (IL-1β), interleukin-8 (IL-8 or CXCL-8), interferon gamma (IFN-γ), interferon-gamma inducible protein 10 (IP-10 or CXCL10), and tumor necrosis factor alpha (TNF-α), induce muscle fiber proteolysis and promote a decrease in protein synthesis, interfering in the myogenic process and disrupting the body homeostasis (Broussard et al., 2004; Pajak et al., 2008; Li et al., 2009; Rayavarapu et al., 2013; Pelosi et al., 2014; VanderVeen et al., 2019; Abdullahi et al., 2020; Cipollaro et al., 2020; Disser et al., 2020; Morley et al., 2020; Sarkesh et al., 2020). Consequently, symptomatic COVID-19 individuals can present musculoskeletal manifestations like fatigue and myalgia due to this uncontrolled inflammatory environment.

Considering these data, we suggest that the systemic release of cytokines, an indirect and well-studied process, is likely the main promoter of the susceptibility of the musculoskeletal tissue to SARS-CoV-2 infection. However, we do not discard the urgent need of more studies to confirm the possibility of a direct binding between the virus and the skeletal muscle cell *via* ACE2 receptor or not. It is noteworthy to highlight that the majority of studies about COVID-19 comprises hospitalized individuals. Thus, some musculoskeletal manifestations can be present due to a prolonged immobilization rather than a direct or an indirect mechanism of action of SARS-COV-2 virus on the muscles.

Additionally, some studies pointed out that the musculoskeletal symptomatology observed in COVID-19 positive patients can be due to pre-existing neuromuscular, muscular or autoimmune disorders, instead of symptoms directly caused by the viral infection. Three studies reported that individuals with myasthenia gravis, a chronic autoimmune disorder, had an exacerbation of their disease (mainly muscle weakness) after SARS-CoV-2 infection (Anand et al., 2020; Paliwal et al., 2020; Ramani et al., 2021). Tseng and Chen also suggested that individuals with motor neuron diseases and dystrophinopathies might be at elevated risk of manifesting exacerbating pre-existing muscle symptoms after COVID-19 infection (Tseng and Chen, 2021). Lastly, myositis, a muscle inflammation that can induce muscle pain and weakness, symptoms observed in COVID-19, was well documented in numerous virus pathologies such as parainfluenza; influenza A/B; hepatitis A, B, C, and E; HIV; Dengue and West Nile, being considered a viral-related disease (Desdouits et al., 2013; Ramani et al., 2021; Wasserman et al., 2021). Although the association “myositis-COVID-19” needs further elucidation, some researchers hypothesized that the muscle symptoms seen in individuals with both diseases can be due to direct viral infection or release of cytokines mediated by the virus (Paliwal et al., 2020; Balcom et al., 2021; Saud et al., 2021; Wasserman et al., 2021); mechanisms that we described in the beginning of this section. We agree with these studies; however, we highlight the urge of more scientific works with detailed data elucidating the involvement of SARS-CoV-2 infection in patients with underlying muscle disorders. As we know, the use of electromyography and other electrodiagnostic and imaging tools can assist on the diagnosis of the myopathic process presented in COVID-19, excluding motor neuron disorders as cause of the observed musculoskeletal symptomatology, as well reported by Ramani et al. (2021).

## Epidemiology and Associated Risks of COVID-19

COVID-19 is transmitted primarily *via* respiratory tract droplets (coughing and/or sneezing), direct contact (human-to-human transmission) and/or indirect contact (contaminated objects and/or surfaces) (Coelho et al., 2020; de Souza et al., 2020; Tu et al., 2020; Zaim et al., 2020; Chilamakuri and Agarwal, 2021). The viral transmission occurs during the pre-symptomatic and symptomatic phases, and even asymptomatic individuals are able to transmit the infection (Romagnoli et al., 2020; Tu et al., 2020; Mehta et al., 2021), which not only results in the fast spread of SARS-CoV-2 in a short period of time, but also creates a considerable difficult in tracing the virus. Studies showed that the average incubation time of SARS-CoV-2 is 1–14 days (Romagnoli et al., 2020; Tu et al., 2020; Zaki and Mohamed, 2021), supporting a quarantine period for positive-asymptomatic, symptomatic and exposed individuals.

Interestingly, even though COVID-19 can affect all age groups, the disease evolves to worse outcomes, such as pneumonia, acute respiratory distress syndrome (ARDS) and/or death when associated with several risk factors (Machhi et al., 2020; Mehta et al., 2021). Older people and individuals with comorbidities like chronic respiratory disease, cardiovascular diseases, chronic kidney disease, cancer, type 2 diabetes mellitus, hypertension and obesity (Baj et al., 2020; Burn et al., 2021; World Health Organization, 2021c) are likely to have a severe form of COVID-19, so a continuous medical support is necessary and indispensable.

## THE Musculoskeletal Symptomatology

COVID-19 can be considered the new “Achilles heel” of Science, because it affects people in numerous ways, causing a variety of different symptoms that are difficult to categorize. According to WHO, the most common clinical symptoms associated with COVID-19 are fever, cough, anosmia (loss of smell), ageusia (loss of taste) and tiredness (fatigue) (World Health Organization, 2021d). WHO classified aches and pains, sore throat, headache, diarrhoea, conjunctivitis, and rashes on skin as less typical symptoms (World Health Organization, 2021d). Curiously, the “list” of common symptoms is somewhat discrepant between the published articles and governmental organizations.

On 22 February 2021, the Centers for Disease Control and Prevention (CDC) of the United States Department of Health and Human Services updated in their website^1^, a more generalized list of common COVID-19-related symptoms in comparison with the list presented by WHO^2^. The CDC list includes symptoms such as fever or chills, cough, shortness of breath, fatigue, muscle or body aches, headache, anosmia and ageusia, sore throat, congestion, nausea or vomiting, and diarrhea (Centers for Disease Control and Prevention, 2021), emphasizing the multi-systemic character of the COVID-19 infection.

**FIGURE 3:**
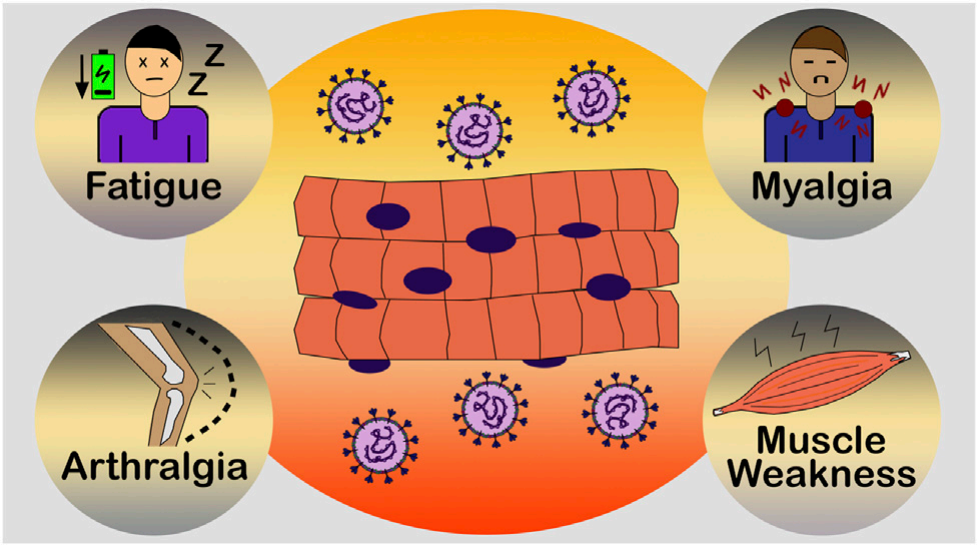
Common muscle symptoms of COVID-19. Fatigue, arthralgia (joint pain), myalgia (muscle pain) and muscle weakness have been reported as initial and common symptoms by SARS-CoV-2-positive individuals. These extenuating symptoms can affect the daily lives of numerous individuals, reducing their quality of life.

Accumulating evidences have showed that the musculoskeletal symptoms can occur during the first days of infection, even before the common respiratory symptomatology (dry cough, nasal congestion, sore throat and dyspnoea). Fatigue, arthralgia (joint pain), myalgia (muscle pain) and muscle weakness have been reported as initial and common symptoms by COVID-19-positive individuals (Figure 3) (Baj et al., 2020; Chan et al., 2020; Cipollaro et al., 2020; Ali and Kunugi, 2021; Kanmaniraja et al., 2021; Knight et al., 2021). Unfortunately, these extenuating symptoms can decrease the individuals’ ability to perform activities of daily living (ADL) such as ambulating, dressing, housecleaning and working (Edemekong et al., 2021), which reduces the quality of life, generating anxiety and depression.

Additionally, it has been well documented case reports of COVID-19 related rhabdomyolysis (Alrubaye and Choudhary, 2020; Chan et al., 2020; Husain et al., 2020; Khosla et al., 2020; Meegada et al., 2020; Mukherjee et al., 2020; Rivas-García et al., 2020; Rosato et al., 2020; Singh et al., 2020; Byler et al., 2021; Haroun et al., 2021; Mah et al., 2021; Patel et al., 2021). Rhabdomyolysis is a skeletal muscle injury that can result in acute kidney injury, a life-threatening clinical complication. It is mainly characterized by elevated levels of creatine kinase (CK; > 200 U/L), a muscular damage marker, myoglobin, potassium and lactate dehydrogenase (LDH) in the bloodstream (Giannoglou et al., 2007; Stahl et al., 2020). As expected, the reported cases associating rhabdomyolysis with SARS-CoV-2 infection comprise only hospitalized patients, which highlights once more, a biased for scientific studies focusing on the severe form of COVID-19.

Furthermore, the different and somewhat discrepant symptoms of COVID-19, including the musculoskeletal ones, difficult the prognostic of the disease, and SARS-CoV-2 viral infection versus musculoskeletal symptomatology is still a subject poorly investigated and a challenge to the researchers in the Muscle Physiology field. Interestingly, some studies highlighted that laboratory findings (elevated levels of CK, LDH, C-reactive protein, creatinine, D-dimer and cytokines; lymphopenia and leukocytosis) and imaging tools (computed tomography—CT scan; magnetic resonance imaging—MRI; positron emission tomography—PET; ultrasound; radiography) can play a crucial role in the prognosis, diagnosis and evaluation of the manifestations of COVID-19, supporting a better treatment of the patients (Feng et al., 2020; Ghayda et al., 2020; Orsucci, 2020; Ponti et al., 2020; Revzin et al., 2020; Afshar-Oromieh et al., 2021; Akbar et al., 2021; Capaccione et al., 2021; Chopra et al., 2021; Khamis et al., 2021; Meng et al., 2021; Ramani et al., 2021; Xie et al., 2021). Unfortunately, the majority of the laboratory and imaging techniques focus on the respiratory, cardiac, gastrointestinal and neurologic systems, and few findings are related to the musculoskeletal apparatus. Even so, we emphasise that the combination of different tools can also contribute to assess the extent and severity of the muscle injury caused by SARS-CoV-2 infection, providing advancements not only on the prognosis of the disease, but on the creation of rehabilitation programs comprising effective physical therapy treatments.

## Long-COVID and Musculoskeletal Sequelae

Recent studies have suggested that the musculoskeletal symptoms, along with the neurological manifestations, prevail after the acute phase of infection, persisting for weeks and/or months, and giving rise to a debilitating condition named as long-COVID (Brüssow and Timmis, 2021; Crook et al., 2021; Fernández-de-las-Peñas et al., 2021; Marshall, 2021; Salamanna et al., 2021). While we are fighting COVID-19, numerous patients around the world, who have suffered from the disease, are calling themselves as “long-haulers,” that is, individuals who present post-COVID symptoms that are lasting even after recovery and viral elimination (Callard and Perego, 2021; Crook et al., 2021; Davis et al., 2021; Fernández-de-las-Peñas et al., 2021).

According to the guidelines of the National Institute for Health and Care Excellence (NICE) of the United Kingdom, long-COVID is defined as “signs and symptoms that continue after acute COVID-19, persisting for more than 4 weeks” (National Institute for Health and Care Excellence, 2020; Sivan and Taylor, 2020; Salamanna et al., 2021). These long-term effects of COVID-19 are usually divided into two categories: (i) “continuous or ongoing symptomatic COVID-19,” which indicates symptoms lasting from 4 to 12 weeks; and (ii) “post-COVID-19 syndrome,” comprising signs and symptoms that persist beyond 12 weeks and are not elucidated by an alternative diagnosis except COVID-19 (National Institute for Health and Care Excellence, 2020; Akbarialiabad et al., 2021).

Interestingly, while Ghosn et al. reported that 60% of the individuals hospitalized for severe COVID-19 in a French cohort study complained of symptoms after 6 months of hospital admission, Carvalho-Schneider et al. observed that even non-critical patients declare to have post-COVID symptoms (Carvalho-Schneider et al., 2021; Ghosn et al., 2021). This observation highlights the need of follow-up studies that include individuals who had mild to moderate COVID-19, and indicates that the post-COVID symptomatology (long-COVID) does not seem to occur only in people recovering from the severe and critical forms of the disease, as also observed by some reports (Graham et al., 2021; Hoffer, 2021; Logue et al., 2021).

Regarding the risk factors associated with long-COVID, they are not well defined; however, studies have reported that this condition occurs more in female patients and individuals with increased age and body mass index, presenting comorbidities, and with a reduced functional status and physical activity practice (Jacobs et al., 2020; Sudre et al., 2021; Yelin et al., 2021). Additionally, the most prevalent symptoms in long-hauler individuals are fatigue, headache, dyspnea, and anosmia (Stavem et al., 2021; Vanichkachorn et al., 2021; Varghese et al., 2021), which emphasizes a persistent neurological and musculoskeletal symptomatology as previously remarked. Fatigue is considered the musculoskeletal symptom most frequently reported by patients after recovery from COVID-19 (Amenta et al., 2020; Rudroff et al., 2020) while myalgias and arthralgias are also common complaints (Liang et al., 2020).

It has been reported that long-haulers can have sequelae from one or more systems such as pulmonary, cardiovascular, gastrointestinal, renal, neural and musculoskeletal (Leviner, 2021). Studies showed that regarding the musculoskeletal system sequelae, COVID-19 survivors, including those who also had the mild to moderate forms of the infection, can experience exacerbated muscle and joint pain (Elhiny et al., 2021), and intense myalgia (muscle pain) (Aiyegbusi et al., 2021; Carvalho-Schneider et al., 2021). Another sequel is intolerance to physical activities associated with a chronic fatigue condition and a difficulty in returning to a normal daily life (Miyazato et al., 2020; Humphreys et al., 2021). Finally, the pathophysiology of the musculoskeletal complications in long COVID is not well understood, but researchers believe that post-COVID symptoms are associated with a persistent pro-inflammatory syndrome (“cytokine storm”) that contributes to long-term sequelae (Fernández-de-las-Peñas et al., 2021; Peghin et al., 2021; Salamanna et al., 2021). We emphasize that a more detailed understanding of the musculoskeletal sequelae in long COVID will be essential for the adequate treatment of long-hauler individuals in the future.

## THE Lack of Musculoskeletal Data Related to Mild to Moderate Forms of COVID-19

The lack of scientific researches focusing on the musculoskeletal system in mild to moderate COVID-19 individuals is a problem that needs attention. The majority of people with symptomatic COVID-19 develop the mild (40%) or moderate (40%) forms of infection, and only 15% evolve to a severe form requiring oxygen support and hospitalization (Chinese Center for Disease Control and Prevention, 2020; World Health Organization, 2021c). Individuals with mild illness present various symptoms of COVID-19, except shortness of breath, dyspnoea and viral pneumonia, while individuals with moderate disease present clinical signs of pneumonia with an oxygen saturation (SpO_2_) ≥ 90% on room air (sea level) (World Health Organization, 2021c; National Institutes of Health, 2021). Therefore, it was expected a considerable number of published articles having as studied subjects the individuals with mild or moderate COVID-19. However, the reality is quite different and frustrating, and it gets worse if you consider the musculoskeletal aspects of COVID-19 in these symptomatic individuals.

**FIGURE 4:**
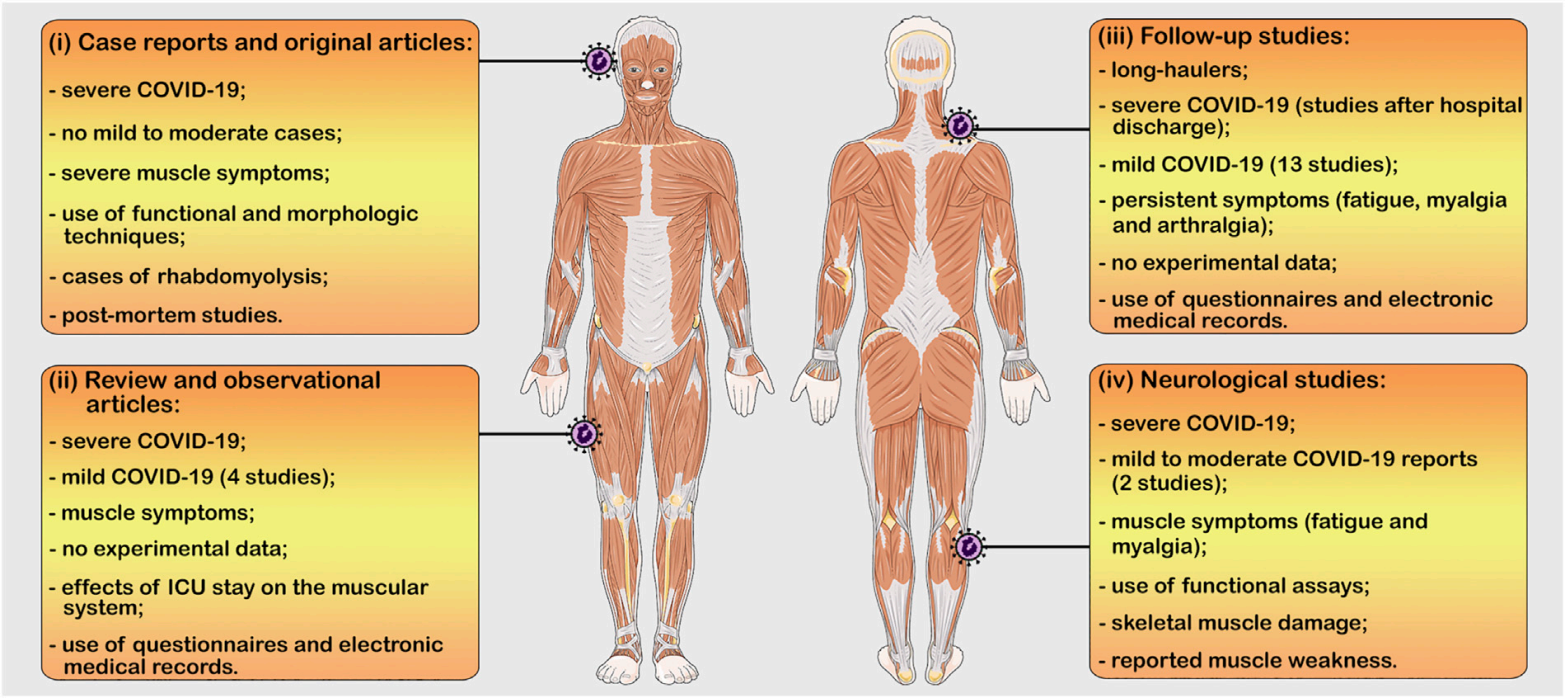
The four main types of studies comprising COVID-19 and the skeletal muscle. (i) case reports and original articles of hospitalized individuals (severe COVID-19; functional and morphologic techniques); (ii) Review and observational articles citing only the musculoskeletal symptoms (mild and severe COVID-19; no experimental data); (iii) Follow-up studies of patients with persistent muscle symptoms (mild and severe COVID-19; no experimental data); and (iv) Neurological studies that includes musculoskeletal symptoms (mild to moderate and severe COVID-19; functional assays). ICU = intensive care unit. Figure produced using Servier Medical Art (smart.servier.com).

We observed, after a comprehensive literature search, the existence of four main types of studies comprising COVID-19 and the skeletal muscle, which we categorize in: (i) case reports and original articles of hospitalized individuals (severe COVID-19); (ii) review and observational articles citing only the musculoskeletal symptoms; (iii) prospective follow-up studies of patients with persistent muscle symptoms (mainly after hospital discharge); and (iv) neurological studies that includes musculoskeletal symptoms (Figure 4).

The case reports and original articles comprised only individuals who were treated in intensive care units (ICU) or received sub-intensive or intermediate care, normally with ventilator support during the diseases’ course. Usually, these studies report the visible muscle wasting of the hospitalized patients (Andrade-Junior et al., 2021; Giraudo et al., 2021; Kumar et al., 2021), as well as musculoskeletal complications such as rhabdomyolysis (Alrubaye and Choudhary, 2020; Zhang Q. et al., 2020; Chan et al., 2020; Khosla et al., 2020; Solís et al., 2020; Byler et al., 2021; Haroun et al., 2021), myalgia (Mukherjee et al., 2020; Uçaroğlu Can et al., 2020; Batur et al., 2021; De Rosa et al., 2021; Vasiliadis et al., 2021), cachexia (Anker et al., 2021), critical illness myopathy (Cabañes-Martínez et al., 2020; Islam et al., 2021), generalized weakness (Chan et al., 2020; Rosato et al., 2020; Paneroni et al., 2021), and increased levels of serum CK (Chan et al., 2020; Khosla et al., 2020; Batur et al., 2021; Byler et al., 2021; De Rosa et al., 2021; Haroun et al., 2021; Orsucci et al., 2021; Pitscheider et al., 2021).

Surprisingly, few studies included functional or morphological techniques in their methodologies. Two scientific works assessed the skeletal muscle strength of hospitalized patients diagnosed with severe COVID-19 through functional tests such as handgrip measurement and maximal voluntary contraction test (dominant *biceps brachii* and *quadriceps*), demonstrating a decrease in muscle strength in the studied individuals (Andrade-Junior et al., 2021; Paneroni et al., 2021). Biopsies of skeletal muscles (*vastus lateralis* and *quadriceps femoris*) were performed in critical and severe cases associated with rhabdomyolysis (Byler et al., 2021; Mughal et al., 2021) and elevated serum CK (Zhang H. et al., 2020; Islam et al., 2021), indicating necrotizing myopathy and myositis. Cabañes-Martínez et al. also confirmed, through muscle biopsy (*quadriceps femoris* and *tibialis anterior*), a diagnosis of critical myopathy in 12 patients with severe COVID-19; however they related this condition to the long stay in ICU (Cabañes-Martínez et al., 2020). Additionally, two studies carry out histological assays in muscle tissues (*rectus abdominis* and *psoas*) of patients, who died from severe COVID-19 (Mageriu et al., 2020; Suh et al., 2021). The authors found signs of necrotizing myopathy, myositis and fiber atrophy in the analysed samples, which, according to them, can be a result of the pro-inflammatory cytokine release that occurs during SARS-CoV-2 infection (Mageriu et al., 2020; Suh et al., 2021). Lastly, a case report described histopathologic alterations (e.g., microthrombi, microhemorrhages, fiber degeneration and necrosis) in the skeletal muscle of a patient who died from COVID-19; however, they associated the observed changes to vascular damage and endothelial injury than to a direct myocite viral infection (Hooper et al., 2021).

The second type of published researches relating COVID-19 to the musculoskeletal system comprised *review and observational articles* briefly describing the muscle symptomatology. These systematic and meta-analysis studies summarize evidences on extrapulmonary features of the infection, aiming to contribute to a better diagnosis, prevention and treatment of COVID-19. Generally, they cited the prevalent muscle symptoms reported by hospitalized patients during the acute phase of SARS-CoV-2 infection (Abdullahi et al., 2020; Ashraf et al., 2020; Small and Beatty, 2020; Zaim et al., 2020; Disser et al., 2020; do Nascimento et al., 2020; Ghayda et al., 2020; Gupta et al., 2020; Kordzadeh-Kermani et al., 2020; White-Dzuro et al., 2021; Kanmaniraja et al., 2021; Ramani et al., 2021; Ramos-Casals et al., 2021), which highlights once more the biased towards severe cases of COVID-19.

Additionally, some studies reviewed the effects of a prolonged hospitalization or ICU stay on the muscular system, focusing on strategies to treat the acquired muscle atrophy seen in the patients (Lad et al., 2020; Sagarra-Romero and Viñas-Barros, 2020; Ali and Kunugi, 2021). Interestingly, three studies summarized evidences that the association between SARS-COV-2, ACE2 and the renin/angiotensin system negatively affects the skeletal muscle, increasing muscle weakness and hindering the full recovery of positive-COVID-19-individuals (Ferrandi et al., 2020; Gonzalez et al., 2020; Yamamoto et al., 2020).

As expected, we only found four studies briefly commenting about the musculoskeletal symptoms (fatigue and myalgia) in mild and non-critically ill cases of COVID-19. Three studies described the clinical characteristics of positive-COVID-19-patients obtained through questionnaires and electronic medical records (Kim et al., 2020; Lechien et al., 2020; Wang et al., 2020), while one study performed a cross-sectional observation that indicated that 5.12% and 2.36% of 254 mild cases of COVID-19 reported myalgia and weakness, respectively (Zahan et al., 2021). No review article comprised studies that applied imaging and/or morphological techniques to evaluate the musculoskeletal manifestations in the mild cases of COVID-19, which emphasizes the lack of essential data that can contribute to a better diagnosis and treatment of these affected individuals.

Following, the third type of research mentioning COVID-19 and skeletal muscle encompassed prospective follow-up studies widely used to identify long-term consequences and persistent symptoms in individuals who had COVID-19. Most studies focus on evaluating patients for weeks or months after hospital discharge (Daher et al., 2020; Jacobs et al., 2020; Chevinsky et al., 2021; Halpin et al., 2021; Leth et al., 2021). As previously mentioned, fatigue, myalgia and arthralgia are the most prevalent musculoskeletal symptoms reported by patients after hospital discharge (severe COVID-19) (Huang et al., 2020; Davis et al., 2021; Ghosn et al., 2021; Logue et al., 2021; Mahmud et al., 2021; Shendy et al., 2021; Sykes et al., 2021; Tolba et al., 2021). They can last for months, resulting in a prolonged muscle weakness and pain that affects the daily lives of the individuals and eventually promotes psychological disorders, such as anxiety and depression.

Unlike the case reports and reviews discussed earlier that mainly focus in evaluate and summarize the musculoskeletal aspects of the severe form of COVID-19, part of the follow-up studies assesses the persistent symptoms, including the muscle ones, in non-hospitalized patients. We found thirteen studies reporting at least one long-term muscle symptom in individuals who had the mild form of the viral infection.

As observed in the severe COVID-19, fatigue was also the prevalent manifestation in mild COVID cases, perduring for almost 2–7 months after the acute onset of the disease and worsening the quality of life of the assessed individuals (Goërtz et al., 2020; Augustin et al., 2021; Moreno-Pérez et al., 2021; Peghin et al., 2021; Shendy et al., 2021). In addition to fatigue, Carvalho-Schneider et al. and Petersen et al. reported the persistence of arthralgia (2 months follow-up), Chopra et al. and Graham et al. reported a prolonged myalgia (2 months follow-up), while Logue et al. reported the presence of muscle aches (9 months follow-up) in mild COVID outpatients (Petersen et al., 2020; Carvalho-Schneider et al., 2021; Chopra et al., 2021; Graham et al., 2021; Logue et al., 2021). Only one study did not report a debilitating long-fatigue, simply observing myalgia and arthralgia in 451 non-hospitalized individuals (Stavem et al., 2021). Tenford et al., on the other hand, followed-up mild cases of COVID-19 for just 21 days after the acute phase, observing a persistence of fatigue in young adults (18–34 years) (Tenforde et al., 2020). The last study comprising mild cases of COVID also reported a prolonged fatigue in 33 patients after 2 months of observation and concluded that the persistent symptoms were not associated with a dysregulated immune response (Fang et al., 2021).

The mentioned studies evaluated their volunteers through electronic medical records, questionnaires, online surveys and/or phone calls, not carrying out any morphological or biochemical assay. They pointed out the need of more data available from mildly symptomatic individuals whose long-term effects of COVID-19 prevail after the end of the infection. We agree that more information and knowledge regarding the musculoskeletal manifestations in non-hospitalized positive-COVID-19 individuals can collaborate with the planning and provision of health services for these patients, and thus allow a better recovery and return to normality.

Lastly, there are studies focusing in the neurological aspects of COVID-19 that includes musculoskeletal symptoms in their findings. This was expected considering the existence of a linkage between the neural (central and peripheral nervous) and the musculoskeletal systems to generate movement (locomotion) (Taga, 1995; Kerkman et al., 2018; Seth et al., 2018). The muscles connected to bones produce movement through contraction and the nervous system controls this movement *via* motor neurons (Kerkman et al., 2018). Additionally, fatigue and muscle pain (myalgia) might result from detrimental changes in the muscle and/or from alterations in the neural input to the skeletal muscle (Mense, 2003; Fitts, 2004).

We observed that several articles within this category reviewed the neurological manifestations and complications of severe COVID-19, reporting skeletal muscle damage associated with myalgia and elevated levels of CK in severely ill patients (Ahmad and Rathore, 2020; Benny and Khadilkar, 2020; Drożdżal et al., 2020; Fotuhi et al., 2020; Khan et al., 2020; Nepal et al., 2020; Pinzon et al., 2020; Puccioni-Sohler et al., 2020; Sheraton et al., 2020; Andalib et al., 2021; Harapan and Yoo, 2021; Moghimi et al., 2021; Orsucci et al., 2021; Quraishi et al., 2021). It was also reported cases of muscle pain associated with muscle denervation atrophy, suggesting Guillain-Barré syndrome, an autoimmune peripheral nervous system disease, as a sequel of the SARS-CoV-2 infection (Aksan et al., 2020; Bahouth et al., 2021; Meyer-Frießem et al., 2021).

The neurological studies that evaluated muscle weakness and pain through functional techniques, such as electroneuromyography (ENMG) and electroencephalography (EEG), showed neuromuscular alterations and the presence of cerebrovascular disease in the inpatients with severe COVID-19 (75% and 3.8%, respectively) along with an ICU-acquired weakness associated with high levels of CK and IL-6 (Karadaş et al., 2020; Bax et al., 2021).

Only two neurological researches explored the neuromuscular effects of COVID-19 in mild to moderate individuals. A follow-up study conducted for 8 months in Denmark, performed electromyography in three muscles (*biceps brachii*, *vastus medialis* and *anterior tibial*) of 20 patients with persistent fatigue (10 individuals presented mild symptoms during the acute phase of disease while 10 individuals had moderate symptoms with an hospitalization stay of 2–9 days) (Agergaard et al., 2021). Myalgia and physical fatigue were the most common reported symptoms (mild-cases: 50% myalgia, 80% physical fatigue; moderate-cases: 30% myalgia, 60% physical fatigue). It was also observed myopathic changes resulting in decreased muscle force in 55% of the individuals, which suggests that myopathy can be a consequence of the exacerbated inflammatory process (high levels of interleukins) promoted by SARS-COV-2 infection even in non-hospitalized individuals (Agergaard et al., 2021). Similarly, a cross-sectional study conducted in Lima (Peru) demonstrated that 46.2% of 199 patients with mild to moderate COVID-19 complained of myalgia and that non-neurological symptoms, such as fever, cough or dyspnea, increased the risk of developing at least one neurological symptom (headache, ageusia, anosmia, dizziness, myalgia) (Garcia et al., 2021).

## Concluding Remarks

Altogether, the few studies that investigated the mild to moderate cases of COVID-19 clearly pointed out that the viral disease affects the musculoskeletal system (acute and long-term effects) even of individuals who did not need hospitalization. However, little is known about the biological and biochemical mechanisms that trigger the muscle symptoms in the mild forms of COVID-19 as most of the available data focus mainly on the muscular manifestations and their consequences after a severe and critical infection that requires internalization and oxygen support. Moreover, the majority of studies investigate only the clinical aspects of COVID-19, carrying out questionnaires, phone calls, and online surveys to summarize the common symptoms of the disease, including the musculoskeletal ones. Analysis of electronic medical records were also realized to associate laboratorial findings and risk factors (e.g., comorbidities) to disease severity and progression. As a result, there is a need for further biophysical and morphological data describing the muscle injury caused by COVID-19 in all positive individuals who experienced some sort of musculoskeletal symptom.

Studies performing biopsy, muscle histopathology, muscle immunofluorescence, skeletal muscle imaging or biochemical techniques are scarce and consequently it is difficult to evaluate if the reported muscle weakness is related to an elevated inflammation (“cytokine storm”), myopathy or other disorder acquire directly or indirectly after SARS-CoV-2 infection. When considering the mild to moderate COVID-19, this difficult only deepens due to the lack of informative studies investigating the natural history of the infection and the cellular and biological mechanisms associated with the common musculoskeletal manifestations (fatigue, myalgia and arthralgia) in the non-hospitalized patients, as utterly highlighted throughout this review.

As the majority of COVID-19 cases comprise mildly symptomatic individuals who can experience persistent muscle symptoms that decrease the ability to perform activities of daily living, it is essential to provide an early diagnosis to these patients, aiming to reduce the risks of viral transmission while supporting them with medical care and rehabilitation services to handle physical and psychological issues.

Hopefully, just as our knowledge about the musculoskeletal system in COVID-19 increases, more studies in this field will be developed in the next months and the care of mild symptomatic patients will be considered a social priority.

## Footnotes

1
www.cdc.gov/coronavirus/2019-ncov/symptoms-testing/symptoms.html.

2
www.who.int/health-topics/coronavirus#tab=tab_3.
